# Respiratory Muscle Strength and Aerobic Performance Among Chronic Obstructive Pulmonary Disease (COPD) Patients: A Correlational Study

**DOI:** 10.7759/cureus.46625

**Published:** 2023-10-07

**Authors:** Mayura P Deshmukh, Tushar J Palekar, Pallavi R Bhakaney, Gaurang Baxi

**Affiliations:** 1 Physiotherapy, Dr. D.Y. Patil Vidyapeeth, Pune, IND

**Keywords:** six-minute walk test, respiratory muscle strength, modified borg scale, microrpm, endurance, chronic obstructive pulmonary disease patients, aerobic capacity

## Abstract

Background

Chronic obstructive pulmonary disease (COPD) is a heterogeneous lung condition, yielding various respiratory symptoms and categorized under several descriptors: early, mild, young, pre-COPD, and preserved ratio impaired spirometry. COPD is synonymous with symptoms such as dyspnea and cough, in addition to others like exercise intolerance, which result from respiratory muscle weakness. Therefore, the emergence of respiratory strength assessment tools for such patients is not surprising. However, evidence is limited regarding the impact of respiratory muscle strength on the physical performance of COPD patients. Therefore, this study employs the MicroRPM device (Medikart HealthCare Systems Pvt. Ltd., Delhi, India) to measure maximum inspiratory and expiratory pressure utilizing mouth pressure.

Methodology

We recruited a total of 40 patients for the study. All patients received a thorough assessment for hemodynamic stability and were categorized according to the Global Initiative for Chronic Obstructive Lung Disease criteria of COPD. The patients then underwent a training session for the MicroRPM device. We took each patient’s inspiratory and expiratory pressure measurements, then determined their six-minute walk distance and modified the Borg scale rating.

Results

We observed no significant correlation between maximum inspiratory pressure (Pimax) and six-minute walk distance (r=−0.023, p=0.890) or modified Borg scale (r=−0.044, p=0.788); additionally, the correlation between maximum expiratory pressure (Pemax) and modified Borg scale was not significant (r=−0.192, p=0.235). However, the correlation between Pemax and six-minute walk distance was both negative and significant (r=−0.384, p=0.014).

Conclusion

Based on our results, respiratory muscle strength can influence the aerobic performance of COPD patients.

## Introduction

Chronic obstructive pulmonary disease (COPD) is defined as “a heterogeneous lung condition characterized by chronic respiratory symptoms (dyspnea, cough, expectoration, exacerbations) due to abnormalities of the airways (bronchitis, bronchiolitis) and/or alveoli (emphysema) that cause persistent, often progressive, airflow obstruction.” The current report outlines new risk factors and categorizations of COPD, including early COPD, mild COPD, young COPD, pre-COPD, and preserved ratio impaired spirometry. The estimated prevalence of COPD in the age group of 5 to 29 years ranges from 0.1% to 0.9%, whereas the incidence ranges from 1.6% to 28.3% in the population above 30 years [1]. Although COPD can be prevented and treated, it can have significant lingering extra-pulmonary effects [2]. As the disease affects the respiratory system, respiratory muscle strength remains an integral part of lung function in such patients [3]. Assessing respiratory muscle strength provides a new perspective on possible causes of unexplained dyspnea and inefficient cough [4]. However, as clinical assessment of respiratory muscle strength is difficult, there is a need for objective quantitative measurements [5]. There are multiple tools for assessing respiratory muscle strength in the literature, which can be categorized as invasive or non-invasive [6]. Although invasive techniques (e.g., esophageal and gastric balloons for recording esophageal, gastric, and trans-diaphragmatic pressure) are considered more reliable, they require complex, long, and unpleasant procedures [7]. Therefore, non-invasive procedures, such as measurement of mouth or nasal pressure, which are easily performed, are usually preferred and are widely applied and accepted [8]. One non-invasive technique is the use of a mouth pressure manometer device called a MicroRPM (Medikart HealthCare Systems Pvt. Ltd., Delhi, India) to measure the maximum inspiratory pressure (MIP) and maximum expiratory pressure (MEP), which, in turn, measures the strength of the respiratory muscles to generate force during a short quasi-static contraction [9,10]. Despite the availability of MicroRPM devices, diagnosis is usually delayed because most screening protocols for dyspnea do not include an assessment of respiratory muscle strength [10]. Therefore, in this study, we aimed to determine respiratory muscle strength and its correlation with endurance in COPD patients using the MicroRPM device.

## Materials and methods

We began this correlational study after receiving approval from the Institutional Ethical Committee of Dr. D.Y. Patil Vidyapeeth Pune (approval number: DYPCPT/ISEC/126/2018). We recruited a total of 40 patients from the hospital for the study. We calculated the sample size using Winpepi software (Version 11.65, J. H. Abramson, August 23, 2016), taking a 95% CI. The patients signed a written informed consent form before participating in the study. We used purposive sampling. Patients diagnosed with COPD based on history, physical examination, radiological findings, and pulmonary function tests were further screened for severity of the disease based on the Global Initiative for Chronic Obstructive Lung Disease criteria. We selected those with mild- or moderate-severity COPD and assessed each individually for hemodynamic stability [4]. Those with co-morbidities and dysfunction of any kind, which would limit their lung performance, were excluded from the study, along with those who were in acute exacerbation of COPD.

Study design and research procedure

This study included 40 patients who were recruited from Dr. D. Y. Patil Hospital and Research Centre, Pune. We used a non-invasive tool, the MicroRPM, to measure the respiratory strength of individual patients. The MicroRPM measured MIP (Pimax) and MEP (Pemax). Measurements occurred with the patient sitting or standing, per his/her comfort. The test is based on measuring the mouth pressure using a manometer attached to a rubber-flanged mouthpiece and a small display showing the results in units of cmH2O. Before the actual measurements, we told the patients to perform inspiratory and expiratory efforts five times as a warm-up [10]. In addition, we attached a nose clip to their nose to avoid any type of air leakage. We asked the patients to perform 18 maximum inspiratory and expiratory efforts in their most comfortable position. Every effort was followed by a 30-second interval. We instructed the patient on using the MicroRPM device and recorded the measurements for analysis. 

## Results

**Figure 1:**
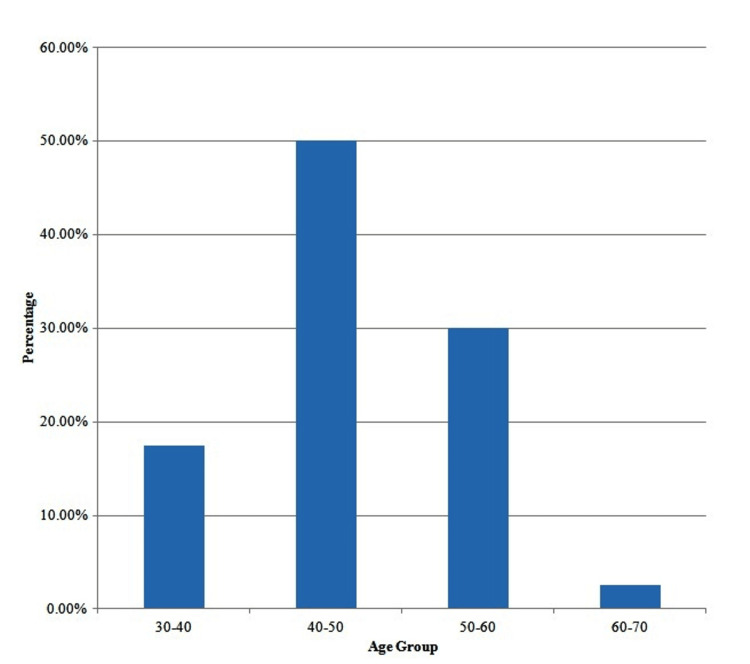
Distribution of patients according to their age in years.

Figure 1 indicates the distribution of patients according to their age in years. Out of 40 patients, seven (17.5%) were in the age group of 30-40 years, 20 (50.0%) were in the age group of 40-50 years, 12 (30.0%) were in the age group of 50-60 years and one (2.5%) was between 60 and 70 years. Figure 3 indicates descriptive statistics for the Pimax and Pemax. The mean Pimax was 79.875 (±11.076) and the mean Pemax was 46.725 (±9.030). 

**Figure 2:**
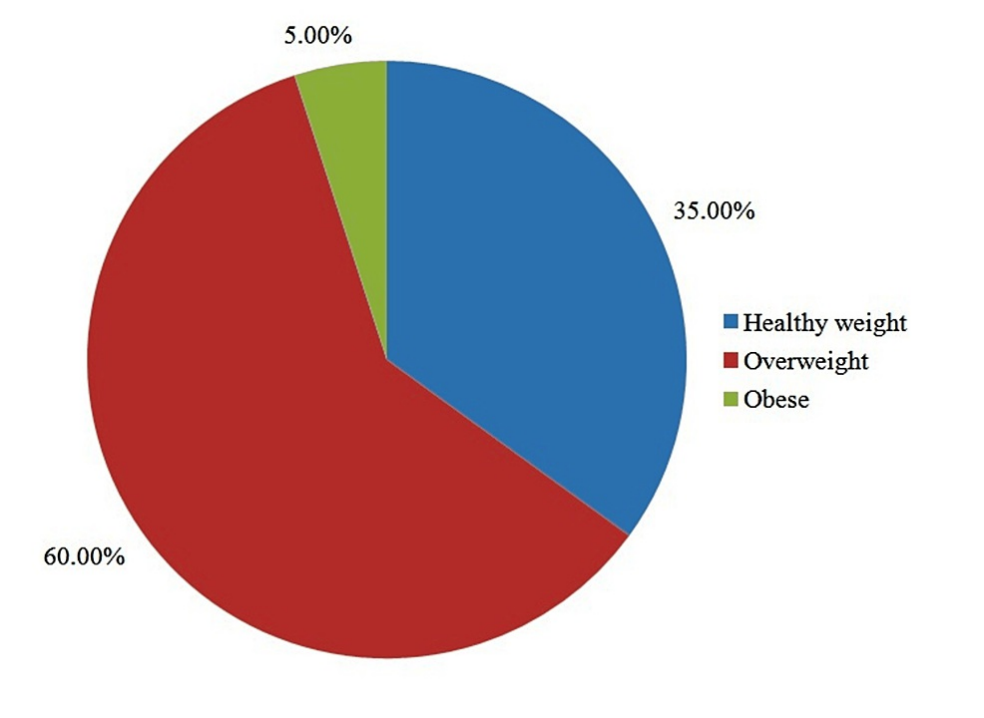
Distribution of study subjects according to their BMI.

Figure 2 indicates the distribution of study subjects according to their BMI. Out of 40 study subjects, 14 (35.0%) were healthy weight, 24 (60.0%) were overweight, and two (5.0%) were obese.

Table 1 indicates a correlation of Pimax and Pemax with distance covered and modified Borg scale. No significant correlation was observed between Pimax and distance covered (r=-0.023, p=0.890) and modified Borg scale (r=-0.044, p=0.788). Additionally, the correlation between Pemax and the modified Borg scale was not significant (r=-0.192, p=0.235). However, the correlation between Pemax and distance covered was negative and significant (r=-0.384, p=0.014).

**Table 1:** Correlation between Pimax and Pemax with distance covered and the modified Borg scale. Correlation is significant at the 0.05 level (two-tailed). Pimax: Maximum inspiratory pressure; Pemax: Maximum expiratory pressure.

		Distance covered	Modified Borg Scale
Pimax cmH2O	Pearson Correlation	-0.023	-0.044
	Significant (two-tailed)	0.89	0.788
	N	40	40
Pemax cmH2O	Pearson Correlation	-0.384*	-0.192
	Significant (two-tailed)	0.014	0.235
	N	40	40
*Correlation is significant at the 0.05 level (two-tailed).			

## Discussion

As illustrated in Figure 1, the majority of COPD patients were between 40 and 50 years old. COPD is prevalent among individuals ≤50 years of age, particularly those with high comorbidities, referred to as young COPD [11]. Figure 2 displays the BMI distribution of the participants: 5% were categorized as obese, 35% as healthy weight, and 60% as overweight. These results are supported by Shinde BV et al., who concluded that the obese population exhibited decreased respiratory muscle strength. The respiratory muscles of such patients are burdened by visceral fat, necessitating greater pressure from the diaphragm for its descent during inspiration [12]. The results of the present study share some similarities with a retrospective study, where authors concluded that patients with acute exacerbation of COPD had reduced inspiratory muscle strength compared with those with stable disease [13], which is why we did not include patients with acute exacerbation of COPD in this study. Another study done by Pereira RN et al. on wheelchair basketball players investigated the relationship between respiratory muscle strength and aerobic performance [14]. There was no significant correlation between the MIP and aerobic performance of the players, as determined by the 12-minute aerobic test. The study results were consistent with those of the present study, where our statistical analysis revealed no correlation between MIP and the aerobic performance measured by the six-minute walk distance test. Cimen B et al. investigated the respiratory muscle strength and aerobic performance of patients affected by rheumatoid arthritis, as well as their clinical and functional correlation. The study concluded that those with rheumatoid arthritis had reduced respiratory muscle strength, endurance, and aerobic capacity [15].

The available literature is not limited to COPD patients. Kim NS et al. investigated lung function and respiratory muscle strength after changes in exercise methods of the diaphragm [16]. Tanriverdi A et al. used a MicroRPM device in their study to determine the effects of high-intensity interval training on patients with heart failure [17]. The results showed that the training improved the patients' quality of life. MicroRPM devices have been widely used in diverse populations to assess respiratory muscle strength. Fuso L et al. used a MicroPRM device to determine the relationship between reduced respiratory muscle strength and endurance in patients with diabetes mellitus. They concluded that the patients who had reduced respiratory muscle strength also had reduced lung volume and rate of metabolic control, and the impaired endurance of the musculoskeletal structures was associated with microvascular complications [18]. The relationship between respiratory muscle strength and relative endurance is not confined to only respiratory conditions. In a study by Katayıfçı N et al., a similar relationship was seen. However, in patients with pacemakers, they concluded that improving respiratory muscle strength enhanced the endurance of such patients [19]. A similar relationship was also observed in patients with neurological disorders [20].

Limitations of the study

This study did have some limitations. First, the study had a small sample size. A larger sample size should be used in the future. Second, we only used two parameters to assess airway obstruction and aerobic capacity. Therefore, additional outcome parameters should be used in future studies to confirm our results.

## Conclusions

We can conclude that there is a prevalence of "young COPD," which necessitates additional attention to manage the disease and enhance the quality of life for these patients. One factor to consider is BMI, which should be acknowledged in the treatment of COPD and other respiratory conditions. Limited studies have established a correlation between respiratory muscle strength and aerobic performance in patients with mild to moderate severity COPD. While no significant differences were observed in the inspiratory strength and aerobic performance of these patients, the findings indicate that respiratory muscle strength does influence aerobic performance in COPD patients with mild to moderate severity. Respiratory muscle strength testing and endurance testing are both safe and effective means of determining a patient's current health and prognosis. In conclusion, it appears vital to incorporate both inspiratory and expiratory muscle training in the rehabilitation protocol for COPD patients.
